# Unusual metastasis from renal cell cancer after partial nephrectomy and sequential targeted therapy

**DOI:** 10.1002/iju5.12261

**Published:** 2021-03-10

**Authors:** Shotaro Yasuoka, Takeshi Yuasa, Motohiro Fujiwara, Junko Fujisaki, Kentaro Inamura, Yoshinobu Komai, Noboru Numao, Shinya Yamamoto, Junji Yonese

**Affiliations:** ^1^ Department of Urology Cancer Institute Hospital Japanese Foundation for Cancer Research Tokyo Japan; ^2^ Department of Gastroenterological Medicine Cancer Institute Hospital Japanese Foundation for Cancer Research Tokyo Japan; ^3^ Department of Pathology Cancer Institute Hospital Japanese Foundation for Cancer Research Tokyo Japan

**Keywords:** bladder metastasis, esophageal metastasis, renal cell cancer

## Abstract

**Introduction:**

Metastatic renal cell carcinoma is treated with various regimens. As their outcomes are improving and follow‐up periods are growing longer, the rate of unusual visceral metastases may increase.

**Case presentation:**

A 68‐year‐old man diagnosed with lung, pancreatic, and renal metastases 9 years after left partial nephrectomy and a diagnosis of pT1a clear cell renal cell carcinoma started molecular targeted therapy using sunitinib. Nine years after the initiation of targeted therapy, a mass lesion in the esophagus was revealed by follow‐up computed tomography, and endoscopic mucosal resection of the esophageal metastatic lesion was performed. One year later, a bladder tumor was detected by follow‐up computed tomography. The patient underwent transurethral resection of the bladder tumor. Histological evaluation of both resected specimens disclosed clear cell renal cell carcinoma.

**Conclusion:**

We present a valuable case of metachronous esophagus and bladder metastases from renal cell carcinoma in a long‐term follow‐up.

**Abbreviations & Acronyms**
CT: computed tomography
IVC: inferior vena cava
RCC: renal cell carcinoma

**Keynote message**

This is the first case of metachronous esophageal and bladder metastasis from RCC. We diagnosed both metastatic lesions by follow‐up CT and treated them endoscopically. As the development of various treatment options continues to increase the survival period for metastatic RCC patients, clinicians must be aware that the rate of metastases in formerly rare sites, including the esophagus and bladder, may increase.

## Introduction

Metastatic RCC is currently treated with various targeted agents and immune checkpoint inhibitors.^1^, ^2^, ^3^ As their overall survival periods getting longer, we may encounter unusual visceral metastases more.^1^, ^2^, ^3^, ^4^ We present a valuable case of esophagus and bladder metastasis from RCC discovered in a routine CT scan during sequential targeted treatments.

## Case presentation

**Fig. 1:**
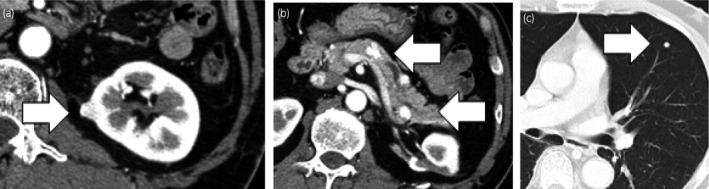
Imaging appearance of the metastatic sites at the initiation of targeted therapy. (a) Ipsilateral renal, (b) pancreas, and (c) lung metastatic lesions at the initiation of targeted therapy.

**Fig. 2:**
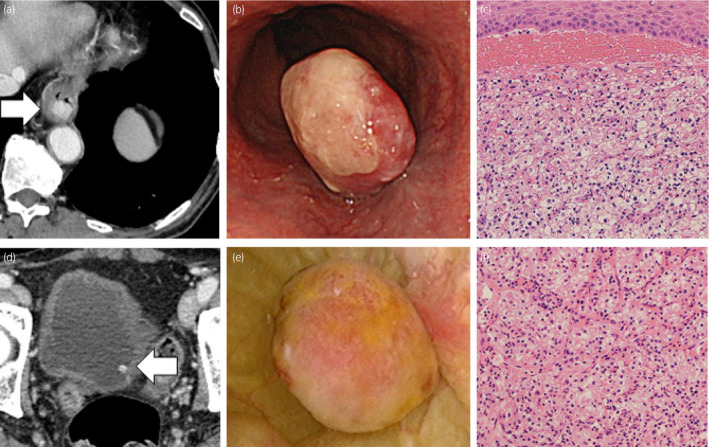
Imaging, endoscopic, and pathological appearance of the esophageal and bladder metastases from renal cell cancer. (a) CT scan revealed a tumor lesion in the esophagus. (b) Endoscopic appearance of a solitary, distinct polypoid esophageal lesion. (d) CT scan revealed a tumor lesion in the bladder. (e) Endoscopic appearance of a solitary, non‐papillary tumor without stalk. Pathological examination of (c) esophageal and (f) bladder lesions disclosed clear cell renal cell cancer.

A 68‐year‐old man diagnosed with ipsilateral renal and multiple pancreatic and lung metastases on annual follow‐up CT scan started molecular targeted therapy using sunitinib (Fig. 1a–c). Nine years earlier, he had undergone left open partial nephrectomy with intraoperative temporary left renal artery ischemia; clinical stage and pathological findings were cT1aN0M0 and pT1a with clear cell RCC, Fuhrman grade 2 and resection margin negative, respectively. Six years after surgery, ipsilateral renal and multiple pancreatic and lung metastases were revealed by annual follow‐up CT scan and he started molecular targeted therapy using sunitinib. He was treated by sequential targeted therapy consisting of sunitinib, everolimus, and axitinib for 3.8, 0.9, and 4.3 years, respectively. Although these treatments showed partial response, those metastatic lesions were growth slowly. Nine years after the initiation of targeted therapy, a mass lesion in the esophagus and extension of the venous thrombus into the IVC from ipsilateral renal recurrent lesion were revealed by follow‐up CT, and upper gastrointestinal endoscopy disclosed a solitary tumor at the lower esophagus (Fig. 2a,b). Endoscopic mucosal resection of the esophageal metastatic lesion was performed. In order to avoid extension of the tumor IVC thrombus, he restarted axitinib therapy. One year later, a bladder tumor was detected by follow‐up CT scan, and cystoscopy revealed a non‐papillary tumor without stalk in the trigon side of his bladder (Fig. 2d,e). He underwent transurethral resection of the bladder tumor. Histological evaluation of both resected specimens disclosed clear cell RCC histology (Fig. 2c,f). Despite restarting axitinib therapy, the patient died due to disease progression 7 months after transurethral resection, 13 years from the first diagnosis of metastasis.

## Discussion

Metastatic RCC to esophageal is extremely rare. At present, 10 cases, including this case, have been reported. Trentino *et al*. reported the first case of clear cell carcinoma of the kidney metastatic to the esophagus.^5^ Dysphagia and dark tarry stools were reported as the presenting symptoms, which were triggers for diagnosis.^6^, ^7^, ^8^, ^9^, ^10^ In our case, follow‐up CT revealed esophageal metastasis without symptoms (Fig. 2a,b). Diagnosis was confirmed by endoscopy and histopathology. There are various treatment options for metastatic esophageal metastasis from RCC. When there is a resectable solitary metastasis lesion, endoscopic or surgical resection is the first choice of treatment. Padda *et al*. reported a case treated endoscopically, and Izumo *et al*. reported a case treated with esophagectomy as surgical resection.^7^, ^11^ In case the tumor is unresectable, molecular‐targeting therapy agents such as tyrosine kinase inhibitors, immune checkpoint inhibitor therapy, and radiation therapy might be administered.

Cabezas‐Camarero *et al*. reported a 38‐year‐old man with esophageal metastasis from RCC and a 4‐year history of metastatic RCC that was treated with combined use of Pazopanib and radiation therapy.^8^ Ali *et al*. reported an 82‐year‐old man with esophageal metastasis from RCC 13 years after nephrectomy that was treated with combined use of sunitinib and radiation therapy.^9^

Bladder metastasis from RCC is also rare. It was reported that 70% of patients with bladder metastasis from RCC experienced hematuria.^12^ There were no specific findings in cystoscopy, and a nodular mass lesion and atypical bladder tumor appearance were reported.^13^ The median time for metachronous bladder metastasis following the diagnosis of RCC was 33 months.^14^ Hematogenous metastasis, retrograde spread, and direct intraluminal transit of tumor cells were proposed as the pathologic mechanism underlying the spread of RCC to urinary bladder.^15^ Transurethral resection is usually performed, and if muscle invasion is revealed pathologically, partial cystectomy is performed.^12^ The prognosis depends on the presence or absence of extravesical metastasis. The 3‐year overall survival rate was around 80% when the metastatic finding was only in the bladder. In contrast, when extravesical metastases coexisted, the 1‐year overall survival was 12.5%.^12^

In conclusion, we reported esophageal and bladder metastasis from RCC. To the best of our knowledge, this is the first case of metachronous esophageal and bladder metastasis from RCC. Both metastatic lesions were detected by follow‐up CT scan during the targeted therapy. As the development of various new effective targeted agents and immune checkpoint inhibitors continues to increase the survival period for metastatic RCC patients, clinicians must be aware that the rate of metastasis in formerly rare sites, including the esophagus and bladder, may increase.

## Conflict of interest

T. Yuasa received remuneration for a lecture from Pfizer Japan (Tokyo, Japan) and Novartis Pharma Japan (Tokyo, Japan).
